# Serum anti-SERPINE1 antibody as a potential biomarker of acute cerebral infarction

**DOI:** 10.1038/s41598-021-01176-8

**Published:** 2021-11-05

**Authors:** Masaaki Kubota, Yoichi Yoshida, Eiichi Kobayashi, Tomoo Matsutani, Shu-Yang Li, Bo-Shi Zhang, Seiichiro Mine, Toshio Machida, Hirotaka Takizawa, Takaki Hiwasa, Yasuo Iwadate

**Affiliations:** 1 Department of Neurological Surgery, Graduate School of Medicine, Chiba University, Inohana 1-8-1, Chuo-ku, Chiba, 260-8670 Japan; 2 Department of Biochemistry and Genetics, Graduate School of Medicine, Chiba University, Chiba, 260-8670 Japan; 3 Comprehensive Stroke Center, Chiba University Hospital, Chiba, 260-8677 Japan; 4 Department of Neurological Surgery, Chiba Prefectural Sawara Hospital, Chiba, 287-0003 Japan; 5 Department of Neurosurgery, Gyotoku General Hospital, Chiba, 272-0103 Japan; 6 Department of Neurological Surgery, Chiba Cerebral and Cardiovascular Center, Chiba, 290-0512 Japan; 7 Department of Neurosurgery, Eastern Chiba Medical Center, Chiba, 283-8686 Japan; 8 Port Square Kashiwado Clinic, Kashiwado Memorial Foundation, Chiba, 260-0025 Japan

**Keywords:** Biomarkers, Neurology, Risk factors

## Abstract

The presence of disease-specific antigens and autoantibodies in the sera of patients with atherosclerosis-related diseases has been widely reported and is considered to result from inflammation of the arterial wall and the involvement of immune factors. The aim of this study was to identify a novel antibody in patients with ischemic stroke by serological identification of antigens using recombinant cDNA expression cloning from patients who had a transient ischemic attack (TIA). We identified the serpin peptidase inhibitor, clade E member 1 (SERPINE1), as a candidate antigen. The serum anti-SERPINE1 antibody levels quantified using amplified luminescent proximity homogeneous assay-linked immunosorbent assay were significantly higher in patients with ischemic stroke, including those with acute cerebral infarction (aCI), TIA, and chronic cerebral infarction, than in healthy donors. The antibody levels were strongly associated with old age, female sex, and presence of hypertension, diabetes mellitus, and cardiovascular disease. Age and intima-media thickness of the carotid artery were positively correlated with antibody levels, which suggests that SERPINE1 may reflect the progression of atherosclerosis. In a multivariate analysis, SERPINE1 antibody level was an independent predictor of aCI. Thus, the serum levels of anti-SERPINE1 antibody could potentially serve as a biomarker of atherothrombotic infarction.

## Introduction

Ischemic stroke has one of the highest morbidity and mortality rates of cerebrovascular disorders worldwide. It is known to be associated with many risk factors such as hypertension, diabetes mellitus (DM), and hyperlipidemia, and individual lifestyle factors such as smoking and drinking habits can also cause ischemic stroke^1^*.* One of the primary pathologies involved in stroke is atherosclerosis. Vascular endothelial cells are affected by risk factors, and atheroma is formed from various immune cells and chronic inflammation, resulting in the progression of atherosclerosis^2^. Antigenic proteins such as oxidized low-density lipoprotein, phosphorylcholine, heat shock proteins, apolipoprotein A1, and phospholipids are involved in this immune response, and the levels of autoantibodies against these proteins are elevated in the sera of patients with atherosclerosis-related diseases such as cerebral infarction, coronary artery disease, and chronic kidney disease^3–5^.

The serological identification of antigens using the recombinant cDNA expression cloning (SEREX) method is useful for identifying antigenic proteins recognized by immunoglobulin G (IgG) antibodies in patients’ sera. Since the report of Sahin et al. in 1995, SEREX has become one of the most effective and accessible methods for identifying tumor antigens in various carcinomas, and > 1000 tumor antigens have been identified using this method thus far^6–10^. SEREX was initially developed to identify tumor antigens, but recently, it has been applied to vascular disorders and autoimmune diseases such as moyamoya disease, Kawasaki disease, systemic lupus erythematosus, and Behcet disease, and is expected to be further developed^11–13^. Previous studies suggested that atherosclerosis is related to immune response-associated inflammation^14–18^. We previously identified RPA2, TUBB2C, ATP2B4, BMP-1, PDCD11, DNAJC2, MMP1, CBX1, and CBX5 as antigens associated with atherosclerotic disease using the SEREX method^19–25^.

In this study, we focused on SERPINE1 (also known as plasminogen activator inhibitor type 1 [PAI-1]), a single-chain glycoprotein of the serine protease inhibitor superfamily, which involves thrombus formation. We identified SERPINE1 antibodies in the sera of patients who had a transient ischemic attack (TIA) and then investigated whether the SERPINE1 antibody could serve as a novel biomarker of ischemic stroke such as acute cerebral infarction (aCI), chronic cerebral infarction (cCI), and TIA.

## Methods

### Serum samples from patients with ischemic stroke and healthy donors

We obtained serum samples from 893 participants, including 612 patients with ischemic stroke and 281 healthy donors (HDs). The patients with cerebral infarction were those diagnosed as having atherothrombotic brain infarction. Patients with ischemic stroke were classified as 459 patients with aCI, 65 patients with cCI, and 88 patients with TIA (Table 1). Serum samples were collected from patients with ischemic stroke from Chiba Prefectural Sawara Hospital, Chiba Rosai Hospital, and Chiba Aoba Municipal Hospital. Serum samples were also collected from HDs from Chiba Prefectural Sawara Hospital and Port Square Kashiwado Clinic. Samples from the enrolled patients were collected upon admission within 2 weeks of onset of ischemic stroke. Individuals with no history of ischemic stroke and no abnormalities on physical examination and brain magnetic resonance imaging were enrolled as HDs. Patients with autoimmune diseases were excluded from the study. The samples were centrifuged at 3000 × *g* for 10 min, and the supernatant was stored at −80 °C until use. Repeated thawing and freezing were avoided.

**Table 1:** Baseline characteristics of the participants.

	HD (n = 281)	aCI (n = 459)	cCI (n = 65)	TIA (n = 88)
Age	54 (23–79)	78 (30–98)**	73 (54–90)**	73 (39–90)**
Male sex (%)	183 (65.1)	268 (58.4)	46 (70.8)	51 (58.6)
Body mass index (kg/m^2^)	23 (15.1–45.7)	22.8 (14.3–39.4)	23.3 (16.4–31.4)	23.5 (15.7–34.2)
Hypertension (%)	57 (20.3)	331 (72.2)**	53 (81.5)**	57 (64.8)**
Diabetes mellitus (%)	10 (3.6)	123 (27)**	22 (33.8)**	26 (29.5)**
CVD (%)	2 (0.7)	39 (8.6)**	2 (3.1)	5 (5.7)*
Hyperlipidemia (%)	40 (14.2)	120 (26.3)**	25 (38.5)**	35 (39.8)**
Smoking (%)	114 (40.6)	227 (49.5)	33 (50.8)	41 (46.6)

Data are medians (interquartile range) for numerical data and n (%) for categorical data.

*CVD* cardiovascular disease, *HD* healthy donor, *aCI* acute cerebral infarction, *cCI* chronic cerebral infarction, *TIA* transient ischemic attack.

*p < 0.01, versus HD; **p < 0.001, versus HD. The Kruskal–Wallis test was performed for continuous variables and the chi-square test was used for categorical variables.

### Clinical data

Data on the age, sex, and risk factors of atherosclerosis, including hypertension, DM, hyperlipidemia, cardiovascular disease (CVD), obesity, and smoking, were collected from the patients’ clinical records. Hypertension was defined as systolic blood pressure > 140 mmHg or diastolic blood pressure > 90 mmHg or a history of antihypertensive drug use. DM was defined as a history of diagnosed diabetes and/or medication and/or fasting blood glucose level of ≥ 126 mg/dl. Hyperlipidemia was defined as a total cholesterol level of > 220 mg/dl, triglyceride level of > 150 mg/dl, or a history of lipid-lowering drug use. CVD was defined as a history of myocardial infarction or angina pectoris. Patients were considered smokers if they smoked or had a history of smoking during the study period. Obesity was defined as a body mass index (BMI) of ≥ 25 kg/m^2^. aCI was defined as the onset of cerebral infarction within 2 weeks. TIA was defined as the presence of a transient episode of neurological dysfunction due to focal cerebral, spinal cord, or retinal ischemia without acute infarction^26^. cCI was defined as cerebral infarction for > 4 weeks.

### Screening by expression cloning

Immunoscreening was performed using modifications of previously published methods^19,21–23,27,28,29^. A commercially available human aortic endothelial cell cDNA expression library (Uni-ZAP XR Premade Library, Stratagene, La Jolla, CA, USA) containing 2 × 10^6^ cDNA clones was used to screen clones for immunoreactivity to serum IgG from 16 patients with TIA. *Escherichia coli* XL-1 Blue MRF′ was infected with Uni-ZAP XR phage and blotted for 2.5 h onto NitroBind nitrocellulose membranes (NitroBind, Osmonics Inc., Minnetonka, MN, USA) that were pretreated with 10 mM isopropyl-β-d-thiogalactoside (IPTG; Wako Pure Chemicals, Osaka, Japan) for 30 min. Membranes deposited with bacterial proteins were washed three times with TBS-T (20 mM Tris-HCl [pH 7.5], 0.15 M NaCl, and 0.05% Tween 20) and incubated with 1% protease-free bovine serum albumin (Nacalai Tesque, Inc., Kyoto, Japan) in TBS-T for 1 h to block nonspecific binding. The membranes were then exposed to the sera of patients who had TIA overnight (1:2000 dilution) and were washed three times with TBS-T again. The membranes were incubated with 1:5000-diluted alkaline phosphatase-labeled goat anti-human IgG (Jackson ImmunoResearch Laboratories, West Grove, PA, USA) for 1 h. Positive reactions were visualized by incubation in a color developing solution (100 mM Tris-HCl [pH 9.5], 100 mM NaCl, and 5 mM MgCl_2_) containing 0.15 mg/ml 5-bromo-4-chloro-3-indolyl phosphate (Wako Pure Chemicals) and 0.3 mg/ml nitroblue tetrazolium (Wako Pure Chemicals). The positive clones were re-cloned twice to obtain monoclonality as described previously^18,21–23^.

### Sequence analysis of the identified antigen

ExAssist helper phage (Stratagene) was used to convert the monoclonal phage cDNA clone into a pBluescript phagemid. The pBluescript plasmid containing the inserted cDNA was obtained from an *E. coli* SOLR strain transformed with the phagemid. The inserted cDNAs were sequenced, and their homology to known genes or proteins was analyzed using the basic local alignment search tool in the RefSeq database of the National Center for Biotechnology Information (http://www.ncbi.nlm.nih.gov/Blast.cgi/).

### Expression and purification of glutathione-S-transferase-tagged antigenic proteins

Recombinant proteins tagged with glutathione-*S*-transferase (GST) were constructed by recombining the cDNA sequence into pGEX-4T-1 vector plasmid (GE Healthcare Life Sciences, Pittsburgh, PA, USA). The pBluescript plasmid containing the cDNA insert was digested with *EcoRI* and *XhoI* and separated using agarose gel electrophoresis. The inserted DNA fragment was isolated using GeneEluteTM Minus EtBr Spin Columns (Sigma-Aldrich, St. Louis, MO, USA), and then ligated in frame to *EcoRI*- and *XhoI*-digested pGEX-4T-1 using the Ligation-Convenience Kit (Nippon Gene, Tokyo, Japan). The ligation mixture was used to transform ECOS competent *E. coli* BL-21 cells (Nippon Gene), and the appropriate recombinant was confirmed on the basis of DNA sequencing and protein expression.

Transformed *E. coli* BL-21 cells containing the pGEX-4T-1 clone were cultured in 200 ml of Luria broth and treated with 0.1 mM IPTG for 3 h. The IPTG-treated cells were harvested, washed with phosphate-buffered saline (PBS), and sonicated in BugBuster Master Mix (Novagen, San Diego, CA, USA) containing 1 mM dithiothreitol. The cell lysates were centrifuged at 13,000 × *g* for 10 min at 4 °C, and the precipitates containing recombinant proteins were dissolved in 8 M urea in TED buffer (50 mM Tris-HCl [pH 8.0], 1 mM EDTA, and 1 mM dithiothreitol). The samples were sequentially dialyzed in steps of 2 h each against 4 and 2 M urea in TED buffer. The samples were then dialyzed using TED buffer dissolved in 0.05 M NaCl for > 12 h and centrifuged at 10,000 × *g* for 30 min at 4 °C. The GST-fused recombinant protein recovered in the supernatant fraction was directly affinity-purified using glutathione Sepharose column chromatography (GE Healthcare Life Sciences) according to the manufacturer’s instructions, and the purified protein was concentrated using Amicon Ultra-15 centrifugal filter equipment (Merck Millipore, Darmstadt, Germany), with removal of glutathione^19,21,24,25,27,30^.

### Western blotting

GST and GST fusion proteins (0.3 μg) were separated using sodium dodecyl sulfate (SDS)-polyacrylamide (11%) gel electrophoresis and transferred to nitrocellulose membranes. The membranes were blocked in 0.5% skim milk powder in a buffer consisting of 20 mM Tris-HCl (pH 7.6), 137 mM NaCl, and 0.1% Tween 20. The blocked proteins were subjected to specific primary antibodies, namely anti-GST antibody (goat; Rockland, Gilbertsville, PA, USA), anti-SERPINE1 antibody (Santa Cruz Biotechnology, Dallas, TX, USA), or 1:5000-diluted sera from the patients or HDs. After incubation with horseradish peroxidase (HRP)-conjugated secondary antibodies (donkey anti-goat or anti-human IgG; Santa Cruz Biotechnology, CA, USA), immunoreactivity was measured using the Immobilon Western HRP Substrate (Merck Millipore) as described previously^10,19,21,27,28,30^.

### Quantification of antibodies using amplified luminescent proximity homogeneous assay-linked immunosorbent assay

Amplified luminescent proximity homogeneous assay-linked immunosorbent assay (AlphaLISA) was used for quantitative measurement of serum antibodies against purified proteins. The indirect format of the conventional competition assay was used in this study. The reaction mixture containing 2.5 μl of serum (1:100 dilution) in AlphaLISA buffer (25 mM HEPES [pH 7.4], 0.1% casein, 0.5% Triton X-100, 1 mg/ml dextran-500, and 0.05% Proclin-300) and 2.5 μl of GST or GST fusion proteins (10 μg/ml) in 384-well microtiter plates (white opaque OptiPlate, Perkin Elmer, MA, USA) was incubated at room temperature for 6–8 h. Then, anti-human IgG-conjugated acceptor beads (2.5 μl, 40 μg/ml) and glutathione-conjugated donor beads (2.5 μl, 40 μg/ml) were added. The mixture was incubated further for 7–21 days at room temperature in the dark. On days 7, 14, and 21, chemiluminescence was read on an EnSpire Alpha microplate reader (Perkin Elmer) as previously described^12,20,21,27,28,31–34^. The results measured on day 14, when the specific response was highest, were used in the analysis. The specific response was calculated by subtracting the alpha value of the GST control (Alpha photon count) from the value of GST-SERPINE1 protein. This alpha value was used as the antibody level reacted with the secondary antibody, the serum antibody of each sample, against 10 μg/ml of GST or GST fusion proteins.

### Quantitative measurement of serum antigen using sandwich enzyme-linked immunosorbent assay

Ninety-six-well polyvinyl chloride microtiter plates were coated with 50 μl of 1.0 μg/ml rabbit anti-PAI-1 antibody (Gene Tex, Irvine, CA, USA) diluted in PBS and kept overnight at 4 °C. The plates were then washed four times with PBS, blocked for 1 h with 50 μl of blocking buffer (10% fetal bovine serum diluted in PBS), and then washed four times with PBS. To obtain a standard curve (calibration curve), purified antigen (10 μg/ml) was prepared by twofold stepwise dilution to a concentration of 100 to 0.2 μg/ml in 50 μl. For measurement of antigen levels, 10 μl of serum was diluted to 50 μl with PBS and incubated for 1 h. The plate was then washed four times with PBS. Fifty microliters of primary antibody anti-SERPINE1 (mouse) diluted 1:2000 in blocking buffer was added to each well. The wells were incubated for 1 h and washed four times with PBS. Similarly, 50 μl of anti-mouse HRP-conjugated secondary antibody (Santa Cruz Biotechnology) diluted 1:5000 in blocking buffer was added to each well. The wells were incubated for 1 h and then washed four times with PBS. One-hundred microliters of chromogenic substrate solution (enzyme-linked immunosorbent assay [ELISA] POD Substrate TMB kit, Nacalai Tesque, Inc., Kyoto, Japan) was added to each well, following by agitation for 30 s and incubation in the dark at room temperature for 10 min. Then, 100 μl of stop solution was added to each well, followed by agitation for 30 s, and the absorbance was immediately measured at 450 nm using a microplate reader (Emax, Molecular Devices, Sunnyvale, CA, USA). A standard curve was prepared by plotting the standard with a series of concentration dilutions on the *x*-axis and absorbance on the *y*-axis. The sample concentration was determined from this standard curve and absorbance value of the sample.

### Immunohistochemical staining

Tissue samples were obtained from surgically resected plaques of nine patients with carotid artery stenosis from Chiba Cerebral and Cardiovascular Center. These patients belonged to a different cohort than the one from which the sera were collected. The samples were fixed in formalin and embedded in paraffin. They were then pretreated by heating in citrate buffer at 98 °C for 40 min. The specimens were incubated with a monoclonal anti-human SERPINE1 antibody (Atlas Antibodies, Stockholm, Sweden), which was used as a primary antibody, at a dilution of 1:100 at 4 °C overnight. The specimens were then incubated with biotin-labeled rabbit anti-mouse/rabbit IgG secondary antibody and then with streptavidin-labeled peroxidase (Dako LSAB 2 System-HRP, Carpinteria, CA, USA). After the 3,3′-diaminobenzidine reaction, as described in the literature, the sections were counterstained with hematoxylin^32^.

### Statistical analyses

All statistical analyses were performed using JMP Pro 14.2.0 software (SAS Institute Inc., Cary, NC, USA). The Kruskal–Wallis test was performed for continuous variables as appropriate. The chi-square test was used for categorical variables. Univariate and multivariate logistic regression analyses were performed to identify a set of variables to classify the participants into those with and those without a history of stroke. The significance of differences among the HD, aCI, cCI, and TIA groups was analyzed using Dunn’s multiple comparison test with type I error adjustment using the Bonferroni procedure. Correlations between the SERPINE1 antibody levels and the individual data of each clinical parameter were evaluated using Spearman correlation analysis. The cutoff SERPINE1 antibody level for predicting ischemic stroke was assessed to maximize the sum of the sensitivity and specificity rates using a receiver operating characteristic (ROC) curve analysis. All the tests were two-tailed, and a *p* value of < 0.05 was considered significant.

### Ethics declarations

This study was approved by the ethics review committees of the Graduate School of Medicine of Chiba University (No. 2017-251) and the cooperating hospital and conducted in accordance with the principles of the Declaration of Helsinki. Written informed consent was obtained from all the participants. The recombinant DNA research was conducted in accordance with the regulations of the Japanese government with official permission from the Graduate School of Medicine, Chiba University.

## Results

### Identification of SERPINE1 by SEREX screening

Immunological screening was performed using sera from the patients in the TIA group, and using SEREX, the SERPINE1 (accession No. NM_000602) clone was identified. The region of SERPINE1 between amino acids 171–689 was obtained as a pBluescript II clone and recombined into pGEX 4T-1 expression vector. The recombinant SERPINE1 protein was expressed as a GST fusion protein in *E. coli* and purified by affinity chromatography using glutathione-Sepharose (Supplementary Fig. S1).

### Presence of autoantibodies against purified proteins in the sera of the patients who had TIA and aCI

**Figure 1:**
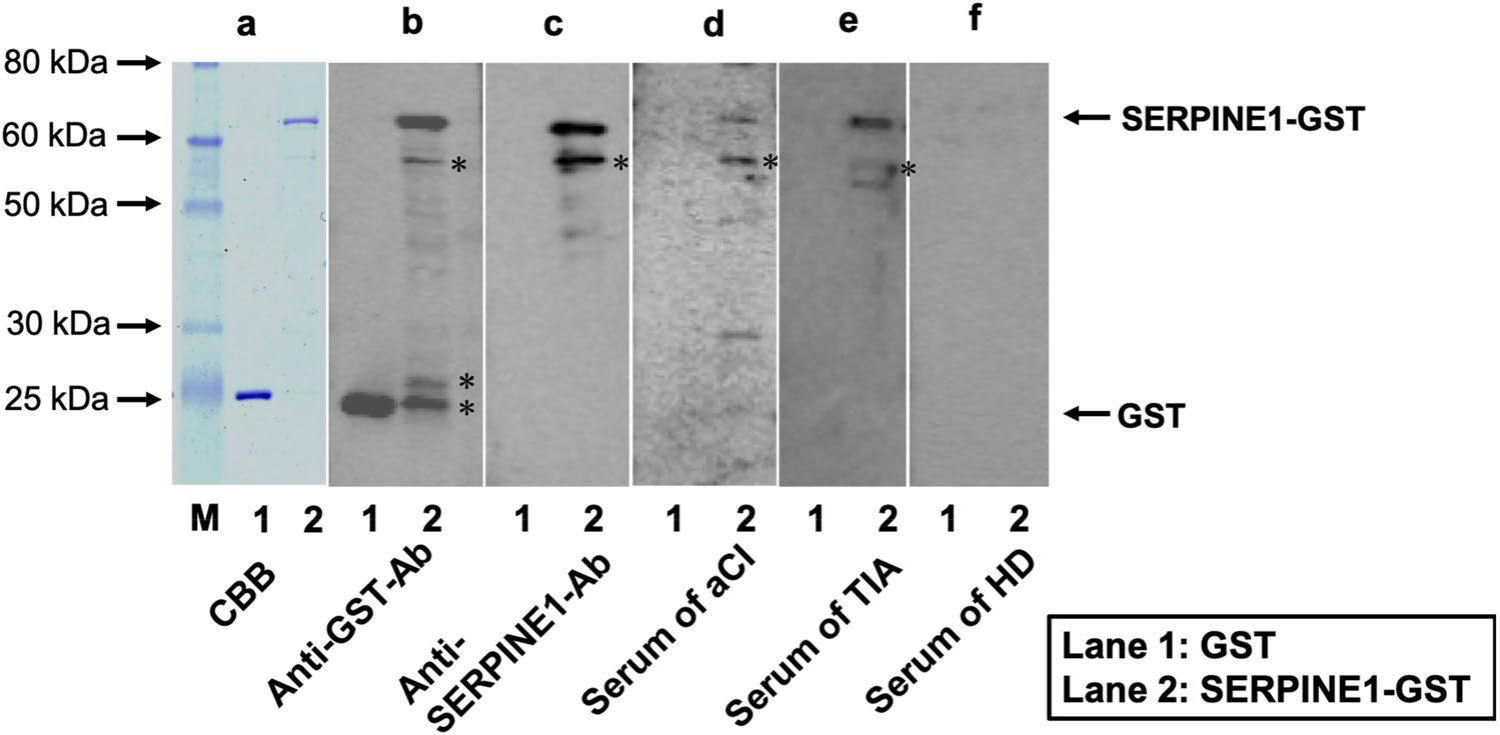
Western blot analysis. Affinity-purified glutathione-*S*-transferase (GST; lane 1) and GST-tagged full length SERPINE1 proteins (lane 2) were electrophoresed with sodium dodecyl sulfate–polyacrylamide (11%) gels, followed by staining with Coomassie Brilliant Blue. (**a**). Western blotting using anti-GST antibody (**b**) and anti-SERPINE1 antibody (**c**). Serum samples of patients with acute cerebral infarction (#7074) (**d**), a transient ischemic attack (#7096) (**e**), and of a healthy donor (#7021) (**f**). Electrophoresed molecular weight markers are also shown in (**a**) (lane M), and the sizes are shown in the left. The asterisk represents degradation products of SERPINE1 after electrophoresis. The number after # is the anonymized patient number and indicates which patient’s serum was used.

Western blotting was performed to demonstrate the presence of anti-SERPINE1 antibody in the serum samples from HDs (#7021) and patients in the aCI (#7074) and TIA groups (#7096). The results showed that GST-SERPINE1 protein detected by anti-GST and anti-SERPINE1 antibodies was recognized by serum antibodies from the patients with aCI and those who had TIA, but not by those from HDs, thereby demonstrating the presence of anti-SERPINE1 antibodies in the sera of patients with stroke (Fig. 1, Supplementary Fig. S2). On the other hand, GST alone did not react to the serum antibodies from HDs or patients with aCI and those who had TIA. The number after # is the anonymized patient number and indicates which patient’s serum was used.

### Elevation of SERPINE1 antibody levels in the patients with ischemic stroke

**Figure 2:**
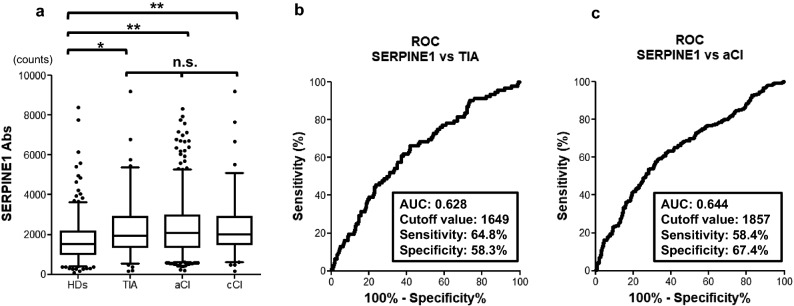
Comparison of serum SERPINE1 antibody levels between the HDs and the ischemic groups. (**a**) Serum antibody levels against SERPINE1 protein quantified using amplified luminescence proximity homogeneous assay-linked immunosorbent assay (AlphaLISA) and compared between the HDs and the patients in the TIA, aCI, and cCI groups. The Alpha photon counts represent the antibody levels and are shown using a box-whisker plot. Horizontal lines represent medians, and boxes represent the 25th and 75th percentiles. Whiskers represent the 10th and 90th percentiles, and dots represent outliers. Differences were examined using Dunn’s multiple comparison test with type I error adjustment using the Bonferroni procedure. HDs, healthy donors; TIA, transient ischemic attack; aCI, acute cerebral infarction; cCI, chronic cerebral infarction; *p < 0.05; **p < 0.001; n.s., not significant. The antibody level of SERPINE1 was analyzed on the basis of the receiver-operating characteristic (ROC) curve for comparison between TIA (**b**) and aCI (**c**). The numbers in the figures indicate the area under the curve (AUC), cutoff values, sensitivity, and specificity.

To investigate the relationship between SERPINE1 antibodies and ischemic stroke, we determined the serum SERPINE1 antibody levels of the patients in the aCI, cCI, and TIA groups and HDs using AlphaLISA. The TIA (*p* = 0.0008), aCI (*p* < 0.0001), and cCI groups (*p* < 0.0001) had significantly higher serum antibody levels than the HD group. This suggests that the antibody levels were maintained not only in the acute phase but also in the chronic phase of ischemic stroke. On the other hand, no significant difference in antibody levels was found among the three stroke groups (TIA, aCI, and cCI groups), which confirms that serum antibody levels are elevated in association with ischemic stroke (Fig. 2a).

### ROC analysis of SERPINE1 antibody levels in the TIA and aCI groups

We performed ROC analysis to determine whether SERPINE1 antibody levels could be used to detect TIA and aCI; thus, cCI was excluded from this analysis. The areas under the curve (AUC) for TIA and aCI were 0.628 and 0.644, respectively, and the cutoff SERPINE1 antibody levels were 1649 counts and 1857 counts, respectively (Fig. 2b, c). When these values were applied, the positive predictive values of the antibody levels alone for TIA and aCI were 32.4% and 74.5%, respectively (Table 2).

**Table 2:** Positive predictive value for TIA and aCI.

	TIA	HDs	Total	PPV	Specificity
SERPINE1-Ab ≥ 1649	57	119	176	32.40% (*p* < 0.0001)	57.7%

	aCI	HDs	Total	PPV	Specificity
SERPINE1-Ab ≥ 1857	271	93	364	74.50% (*p* < 0.0001)	66.9%

The positive predictive values and specificity for TIA and aCI were calculated using the SERPINE1 cutoff values obtained from the receiver operating characteristic curve analysis.

*TIA* transient ischemic attack, *HDs* healthy donors, *PPV* positive predictive value, *aCI* acute cerebral infarction, *Ab* antibody.

### Correlation analysis between SERPINE1 antibody level and clinical features in the validation cohort

We performed Spearman correlation analysis to identify if there was a relationship between the SERPINE1 antibody levels and the clinical features of 893 samples in the validation cohort (Table 3). Patient information included hematological examination results, lifestyle habits, and physical findings such as age, sex, height, weight, BMI, and intima-media thickness (IMT) of the carotid artery. The correlation analysis revealed positive correlations between the SERPINE1 antibody levels and age (*r* = 0.2308, *p* < 0.0001), IMT of the right carotid artery (*r* = 0.2263, *p* < 0.0001), IMT of the left carotid artery (*r* = 0.1974, *p* < 0.0001), and maximum IMT (*r* = 0.2231, *p* < 0.0001). The SERPINE1 antibody levels were also correlated with height, weight, total bilirubin level, albumin level, white blood cell count, red blood cell count, and blood glucose levels. Lifestyle habits such as alcohol drinking frequency and smoking period were not associated with the antibody levels.

**Table 3:** Spearman correlation analysis between the serum SERPINE1 antibody levels and clinical features.

Variable	Spearman rank correlation coefficient (r)	*p* value
Age	0.2308	**< 0.0001**
Height	0.0975	**0.0037**
Weight	− 0.1306	**0.0001**
BMI	− 0.0386	0.2562
Right IMT	0.2263	**< 0.0001**
Left IMT	0.1974	**< 0.0001**
Maximum IMT	0.2231	**< 0.0001**
AST	− 0.0319	0.4139
ALT	0.0178	0.6488
ALP	0.0684	0.0934
LDH	− 0.0028	0.9442
Tbil	− 0.0798	**0.043**
CHE	− 0.0537	0.239
γ-GTP	0.071	0.0794
TP	− 0.0502	0.2058
ALB	− 0.1083	**0.0059**
BUN	0.034	0.3841
Creatinine	0.032	0.413
eGFR	− 0.0509	0.214
UA	0.0014	0.9753
AMY	0.0018	0.9722
T-CHO	− 0.0251	0.5509
HDL-C	− 0.0077	0.878
TG	− 0.042	0.3827
Na	0.0491	0.2124
K	− 0.0461	0.2417
Cl	0.0689	0.0801
CRP	0.0486	0.3003
WBC	0.1075	**0.0058**
RBC	− 0.0962	**0.0136**
HGB	− 0.0741	0.0575
HCT	− 0.0762	0.0509
PLT	− 0.0045	0.9075
Blood glucose	0.0804	**0.0482**
HbA1c	0.0304	0.5008
Smoking period	0.0372	0.3433
Alcohol frequency	− 0.0402	0.326

A correlation analysis was performed to identify the relationship between SERPINE 1 antibody levels and the clinical features of the validation cohort.

Correlation coefficient (*r*) and *p* values were calculated using Spearman correlation analysis. Significant correlations are marked in bold.

*BMI* body mass index, *IMT* intima-media thickness, *AST* aspartate aminotransferase, *ALT* alanine aminotransferase, *ALP* alkaline phosphatase, *LDH* lactate dehydrogenase, *Tbil* total bilirubin, *CHE* choline esterase, *γ-GTP* gamma-glutamyl transpeptidase, *TP* total protein, *ALB* albumin, *BUN* blood urea nitrogen, *eGFR* estimated glomerular filtration rate, *UA* uric acid, *AMY* amylase, *T-CHO* total cholesterol, *HDL-C* high-density lipoprotein cholesterol, *TG* triglyceride, *CRP* C-reactive protein, *WBC* white blood cell count, *RBC* red blood cell count, *HCT* hematocrit, *PLT* platelet count, *HbA1c* hemoglobin A1c.

**Figure 3:**
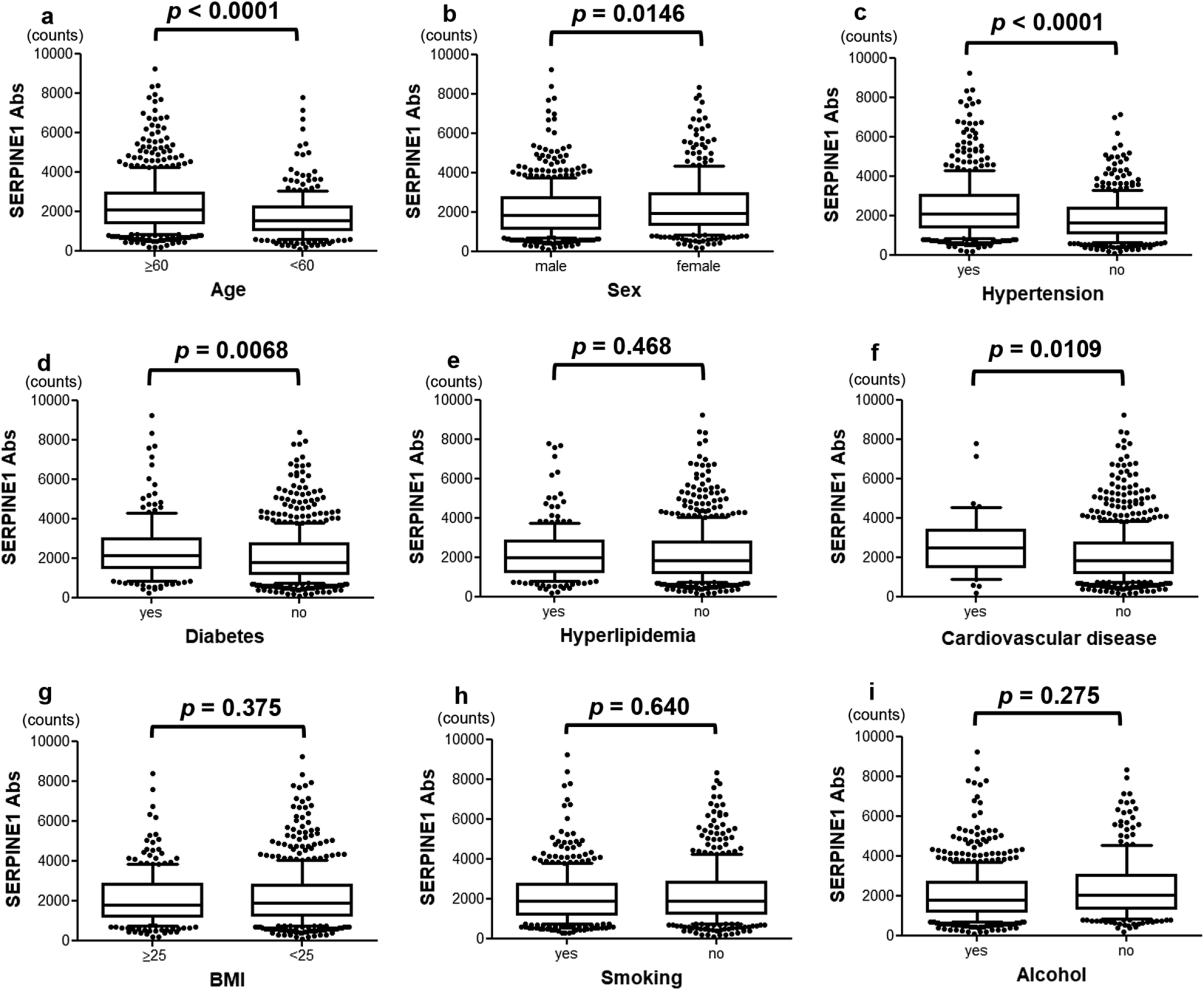
Association between SERPINE1 antibody levels and clinical data. The associations between SERPINE1 antibody levels and clinical parameters such as old age (**a**); sex (**b**); hypertension (**c**), diabetes (**d**), hyperlipidemia (**e**), cardiovascular disease (**f**); body mass index (BMI) (**g**); history of smoking (**h**); and alcohol consumption (**i**) were examined. The SERPINE1 antibody levels determined using AlphaLISA are shown in the box-whisker plots. The *p* values were calculated using the Kruskal–Wallis test.

Clinical factors such as old age, sex, medical history, obesity, and histories of smoking and alcohol consumption were examined in relation to serum SERPINE1 antibody levels. The antibody levels were significantly elevated in patients aged ≥ 60 years (*p* < 0.0001), female patients (*p* = 0.0146), and those with a history of hypertension (*p* < 0.0001), DM (*p* = 0.0068), or CVD (*p* = 0.0109). On the other hand, there was no significant difference in SERPINE1 antibody level regarding other parameters, such as obesity and histories of smoking and alcohol consumption (Fig. 3).

Using the clinical risk factors and cutoff values obtained earlier, univariate logistic regression analysis for the TIA group revealed significant differences in age ≥ 60 years (*p* < 0.0001), hypertension (*p* = 0.0003), DM (*p* = 0.0008), and hyperlipidemia (*p* = 0.0289) but not for antibody levels (*p* = 0.0587; Table 4). Similar results were obtained when SERPINE1 antibody levels were analyzed as a continuous variable per 100 counts (Supplementary Table S1). On the other hand, univariate logistic regression analysis in the aCI group revealed that not only clinical risk factors such as age ≥ 60 years (*p* < 0.0001), hypertension (*p* < 0.0001), DM (*p* < 0.0001), and hyperlipidemia (*p* = 0.0374) but also an antibody level of ≥ 1857 counts (*p* = 0.0155) were associated with aCI. Multivariate analysis of the univariate data with *p* values of < 0.05 revealed that in addition to the atherosclerotic factors mentioned earlier, elevated SERPINE1 antibody level was an independent predictor of aCI (odds ratio [OR]: 1.76, 95% confidence interval [CI]: 1.15–2.68, *p* = 0.0088; Table 5). These results remained unchanged when SERPINE1 antibody levels were analyzed as a continuous variable per 100 counts (Supplementary Table S2).

**Table 4:** Logistic regression analysis of the predictors of transient ischemic attack.

	Univariate analysis			Multivariate analysis		
	OR	95% CI	*p* value	OR	95%CI	*p* value
Age ≥ 60 years	4.7	2.45–9.07	**< 0.0001**	5.34	2.84–10.07	**< 0.0001**
Male sex	0.94	0.46–1.91	0.8672			
HT	3.11	1.67–5.78	**0.0003**	3.55	1.96–6.44	**< 0.0001**
DM	4.6	1.85–11.45	**0.0008**	4.52	1.87–10.93	**0.0006**
HL	2.15	1.09–4.23	**0.0289**	1.85	0.95–3.56	0.0695
CVD	1.44	0.23–9.12	0.6938			
BMI ≥ 25 kg/m^2^	0.77	0.39–1.51	0.4542			
Smoking	0.93	0.46–1.83	0.8241			
SERPINE1-Ab ≥ 1649	1.81	0.98–3.36	0.0587			

The SERPINE1 antibody cutoff value of 1649 counts based on the ROC curve analysis for the TIA group was applied in the univariate analysis.

Univariate data with *p* values of < 0.05 were included in the multivariate analysis. *p* values of < 0.05 are marked in bold.

*HT* hypertension, *DM* diabetes mellitus, *HL* hyperlipidemia, *CVD* cardiovascular disease, *BMI* body mass index, *OR* odds ratio, *CI* confidence interval, *Ab* antibody.

**Table 5:** Logistic regression analysis of predictors of acute cerebral infarction.

	Univariate analysis			Multivariate analysis		
	OR	95% CI	*p* value	OR	95%CI	*p* value
Age ≥ 60 years	13.14	8.28–20.83	**< 0.0001**	14.18	9.05–22.19	**< 0.0001**
Male sex	0.95	0.57–1.60	0.8663			
HT	5.74	3.67–9.00	**< 0.0001**	5.48	3.54–8.48	**< 0.0001**
DM	7.48	3.34–16.76	**< 0.0001**	8.39	3.36–16.24	**< 0.0001**
HL	0.55	0.31–0.97	**0.0374**	0.55	0.32–0.95	**0.0337**
CVD	3.24	0.72–14.59	0.1248			
BMI ≥ 25 kg/m^2^	0.76	0.46–1.23	0.2581			
Smoking	1.19	0.73–1.94	0.488			
SERPINE1-Ab ≥ 1857	1.69	1.11–2.60	**0.0155**	1.76	1.15–2.68	**0.0088**

The SERPINE1 antibody cutoff value of 1857 counts based on the ROC curve analysis for the aCI group was applied in the univariate analysis.

Univariate data with *p* values of < 0.05 were included in the multivariate analysis. *p* values of < 0.05 are marked in bold.

*HT* hypertension, *DM* diabetes mellitus, *HL* hyperlipidemia, *CVD* cardiovascular disease, *BMI* body mass index, *OR* odds ratio, *CI* confidence interval, *Ab* antibody.

On the basis of the multivariate analysis results, we determined the positive predictive value of the three clinical factors (age ≥ 60 years, hypertension, and DM) and the antibody levels, which were significantly associated with the prediction of stroke onset. Combining not only the three clinical factors but also the SERPINE1 antibody levels improved the accuracy of the positive predictive value (Table 6).

**Table 6:** Positive predictive value including the factors associated with the prediction of acute cerebral infarction.

	Clinical risk factors				Clinical risk factors +			
					Anti-SERPINE1-Ab ≥ 1857			
	aCI	HD	PPV (%)	Specificity (%)	aCI	HD	PPV (%)	Specificity (%)
Age ≥ 60 years	408	79	83.8	71.8	252	30	89.4	89.3
HT	331	57	85.3	79.7	200	27	88.1	90.4
DM	123	10	92.5	96.4	73	3	96.1	98.9
Age ≥ 60 years + HT	301	28	91.5	90.0	188	12	94.0	95.7
Age ≥ 60 years + DM	107	4	96.4	98.5	68	1	98.6	99.6
Age ≥ 60 years + HT + DM	89	2	97.8	99.3	58	0	100.0	100.0

Results of the multivariate analysis were used to determine the positive predictive value based on the clinical factors that were particularly relevant.

The numbers under aCI and HD indicate the number of participants.

The left side of the table shows the number of people with only clinical risk factors, whereas the right side shows the number of people with clinical risk factors and anti-SERPINE1 antibody levels above the cutoff value.

The total number of aCI and HD were 459 and 281, respectively.

*HT* hypertension, *DM* diabetes mellitus, disease, *aCI* acute cerebral infarction, *HD* healthy donor, *PPV* positive predictive value, *Ab* antibody.

### Immunohistochemical staining of arteriosclerosis plaque

**Figure 4:**
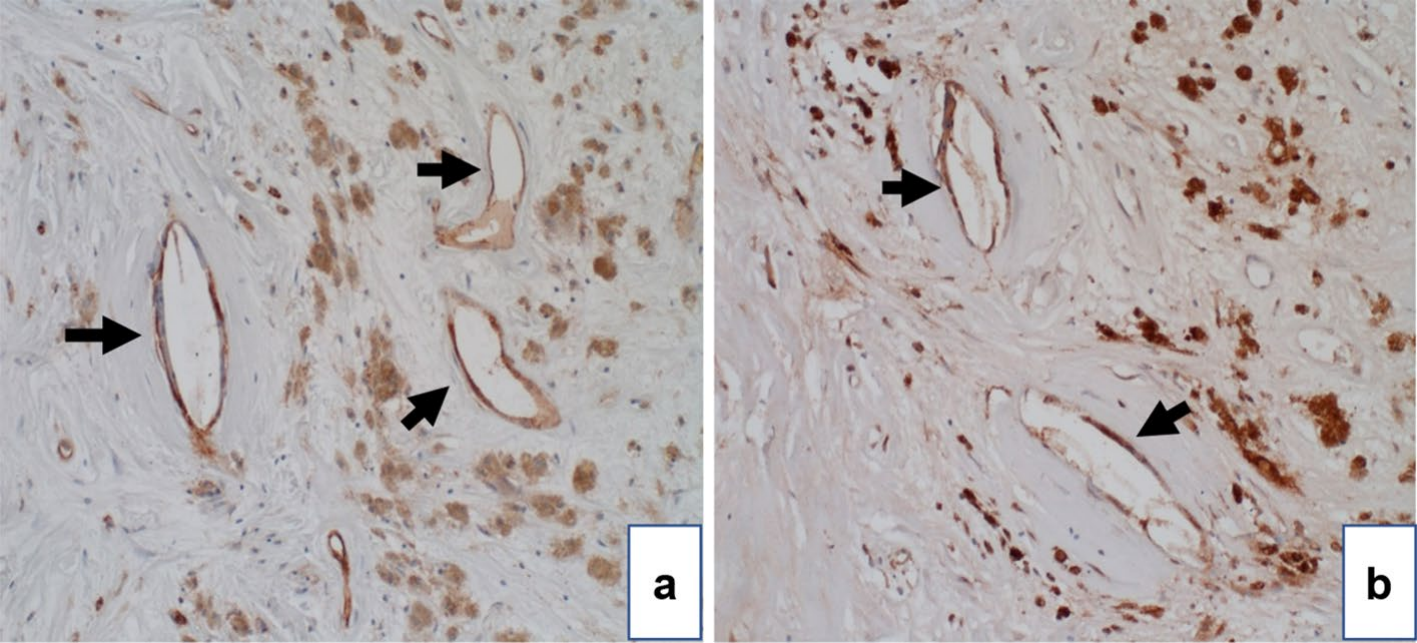
Immunohistochemical staining for SERPINE1 in an arteriosclerotic plaque. Immunohistochemical staining of atherosclerotic plaques samples from nine patients at the Chiba Cerebral and Cardiovascular Center was performed. A representative case is presented here. These samples were obtained from a different cohort than that from which the sera were collected. Atherosclerotic plaque resected during carotid endarterectomy was immunostained using anti-CD31 (**a**) or anti-SERPINE1 antibodies (**b**). Arrows indicate vascular endothelial cells labeled with CD31 antibody, and correspondingly, SERPINE1 was stained by anti-SERPINE1 antibody.

As the SERPINE1 antibody levels were elevated in patients with ischemic stroke and were correlated with carotid IMT, we performed carotid endarterectomy in nine patients with carotid stenosis and immunohistochemical staining of the intima of the resulting carotid sclerotic plaques using anti-SERPINE1 antibody and a vascular endothelial cell marker, anti-CD 31 antibody. Both antibodies exhibited similar staining profiles (Fig. 4a, b), which suggests that SERPINE1 protein was predominantly expressed in vascular endothelial cells.

### Correlation between SERPINE1 antigen and antibody levels

**Figure 5:**
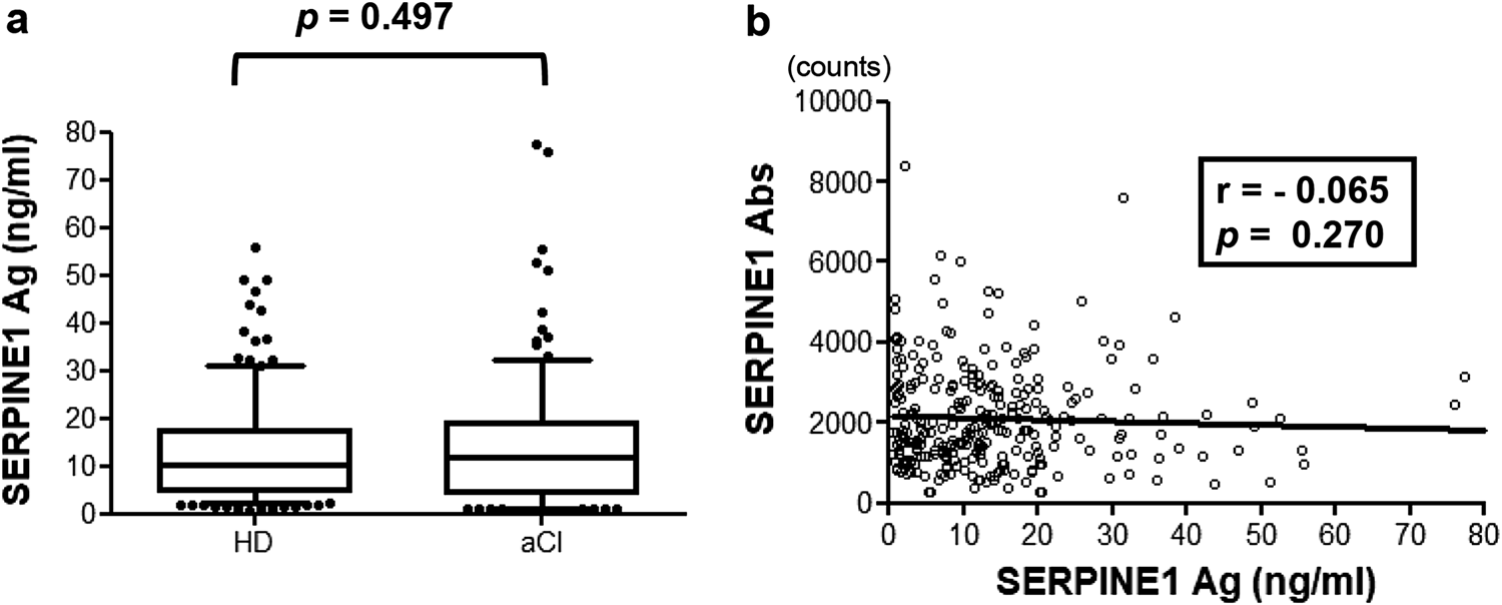
Comparison of serum SERPINE1 antigen levels between the aCI and HDs groups. (**a**) Serum SERPINE1 antigen levels were examined using sandwich enzyme-linked immunosorbent assay in healthy donors and patients with acute cerebral infarction. The results are shown in a box-whisker plot. The *p* values were calculated using the Kruskal–Wallis test. (**b**) SERPINE1 antibody levels were plotted against SERPINE1 antigen levels in a scattered plot. The correlation coefficient (*r* value) and *p* value were calculated using the Pearson product–moment correlation coefficient.

SERPINE1 antigen levels were analyzed in 288 age-adjusted samples (144 samples each for aCI and HDs) using the sandwich ELISA method. A comparison of the antigen levels between the two groups revealed no significant difference (*p* = 0.4974; Fig. 5a). As the SERPINE1 antibody levels of each patient were determined using AlphaLISA, the correlations between SERPINE1 antigen and antibody levels were examined using the linear regression equation, but no correlation was found (*r* =  − 0.065, *p* = 0.270; Fig. 5b). In addition, antigen levels and clinical factors were examined using Spearman’s correlation analysis, but no significant correlation was found (Supplementary Table S3).

## Discussion

Immunological involvement in atherosclerosis has been studied in detail, but autoantibodies against antigens in the sera of patients with atherosclerosis-related diseases have not been fully investigated. SEREX is useful for screening not only for tumor antigens but also for antibody biomarkers of atherosclerotic diseases such as stroke and diabetes. In this study, we employed SEREX to identify the antigen SERPINE1 in the sera of patients with ischemic stroke and confirmed the presence of autoantibodies against SERPINE1 and IgG antibodies using Western blotting (Fig. 1). We quantified the serum SERPINE1 antibody levels in 893 participants using AlphaLISA and evaluated the results between the ischemic stroke and HD groups.

SERPINE1 is a member of the serine proteinase inhibitor superfamily. It is a 45-kDa protein comprising 379 amino acids and is encoded by *SERPINE1* in humans. SERPINE1 is an acute-phase reactive protein in inflammation that is produced by vascular endothelial cells, platelets, megakaryocytes, smooth muscle cells, fibroblasts, macrophages, adipocytes, and cardiomyocytes^35^. High SERPINE1 levels have been reported to be associated with the risk of cardiovascular and atherosclerotic diseases^36–40^. The mechanism by which SERPINE1 forms a complex with tissue-type plasminogen activator (t-PA) and exhibits potent antifibrinolytic activity leading to the promotion of thrombus formation is well known^41^. Therefore, we can infer that people who are chronically exposed to SERPINE1 in conjunction with vascular endothelial damage and inflammation caused by the progression of atherosclerosis are at high risk of thrombosis. These functions suggest that autoantibodies against SERPINE1 may have the ability to inhibit thrombosis.

We performed AlphaLISA to quantify the antibody levels in the serum samples; serum SERPINE1 antibody levels were significantly higher in the patients in the ischemic stroke group than in the HDs (*p* < 0.001; Fig. 2a). No apparent difference was found between the ischemic stroke groups. In the association between antibody levels and clinical factors, significant differences were observed in terms of age > 60 years, female sex, and the incidences of hypertension, DM, and CVD (Fig. 3). To evaluate the ability of SERPINE1 antibodies to detect TIA and aCI, the positive predictive values were calculated using the cutoff value obtained from the ROC curve (Fig. 2b, c), and positive predictive values of 32.4% and 74.5%, respectively, were obtained (Table 2). In the TIA group, univariate logistic regression analysis of the clinical risk factors and antibody levels revealed no significant difference in antibody level, which suggests that the antibody levels cannot be used to predict TIA. In contrast, a significant difference in SERPINE1 antibody levels was observed in the aCI group in addition to the previously reported atherosclerotic risk factors such as old age; presence of hypertension, DM, and hyperlipidemia; and antibody level ≥ 1857 counts, which were identified as independent predictors in the multivariate logistic regression analysis (Table 5)^42–46^. The positive predictive value was calculated based on clinical factors that were significant in the multivariate analysis. The result showed that the inclusion of anti-SERPINE1 antibody as a clinical factor slightly increased the positive predictive value compared with clinical factors alone. These results indicate that immunity to SERPINE1 is a factor that is associated with the progression of atherosclerosis and the development of aCI.

The correlation analysis between SERPINE1 antibody level and the clinical features, including physical and hematological findings, revealed significant positive correlations with age, bilateral carotid IMT, and maximum IMT (Table 3). IMT refers to the thickness of the intima-media complex of the carotid artery as measured on carotid echocardiography. Thickening of the IMT represents the development of atherosclerosis, and its progression can lead to aCI and CVD^47–50^. Therefore, an important finding is that SERPINE1 antibody levels can serve as an indicator of atherosclerosis progression. As SERPINE1 antibody levels were positively correlated with IMT, we examined arteriosclerotic plaques from patients with carotid artery stenosis using immunohistochemistry and found that SERPINE1 protein was highly expressed, consistent with intima of the vascular plaques (Fig. 4). This result supports previous reports of the presence of elevated SERPINE1 expression in atherosclerotic plaques^51–53^. Elevated SERPINE1 antigen expression in atherosclerotic plaques may promote plaque rupture by decreasing the cellular component of the fibrous cap. After plaque rupture, SERPINE1 is suddenly released from the damaged plaque and platelets, increasing its concentration and consequently promoting thrombus formation^54–56^.

On the basis of the results of the immunohistochemical staining of the carotid plaques, we speculated that SERPINE1 antigen may be highly expressed in patients with atherosclerosis progression, which may lead to an increase in the level of SERPINE1 antibodies. However, when the aCI and HDs groups were compared on the basis of the antigen levels determined using sandwich ELISA, no significant difference in antigen level (*p* = 0.4974) and no significant correlation were observed between the antigen and antibody levels (*r* = − 0.065, *p* = 0.270; Fig. 5a, b). Furthermore, the correlation between the antigen levels and other clinical features was examined using Spearman correlation analysis, and factors such as age and IMT showed no significant differences. Consistent with our finding that SERPINE1 antigen levels are not associated with cerebral infarction, previous reports indicated that SERPINE1 antigen levels are not associated with the prediction of CVD or stroke. Other reports controversially discussed the potential role of antigens in myocardial infarction and stroke^57–61^.

Based on our findings, we believe that SERPINE1 antigen level is not a reliable biomarker for ischemic stroke. There are several possible explanations for the lack of correlation of SERPINE1 antigen levels with stroke: (1) SERPINE1 has a short half-life of 90–120 min at 37 °C after release into the bloodstream; (2) the level of the released protein fluctuates with diurnal variation; and (3) SERPINE1 exists with tissue plasminogen activator in the blood in active, latent, and complex forms, and the values can vary depending on the form of the antigen being measured^62–64^. Furthermore, SERPINE1 antigen levels increased immediately after the onset of stroke or immediately after surgery but returned to baseline within 24 h, which suggests that the increase in antigen levels in the acute phase is short-lived^65,66^. Thus, quantitative measurement of SERPINE1 antigen level varies and tends to be more sensitive immediately after a surgery and vascular events, which suggests that it is not suitable for predicting cerebral infarction before the onset or as an indicator of atherosclerosis. Therefore, SERPINE1 autoantibodies, which are amplified by repeated exposure to proteins secreted from the vascular endothelium and plaques even before the onset of vascular events as a result of chronic inflammation associated with atherosclerosis, may be more sensitive as biomarkers than antigens^67^.

Based on our results, SERPINE1 antibody level is a potentially useful biomarker for the detection of aCI and indicator of atherosclerosis. Although the cutoff SERPINE1 antibody level alone did not have high predictive diagnostic accuracy for aCI, the multivariate logistic regression analysis performed to determine the positive predictive value of antibody level in combination with clinical risk factors, which are independent predictors, improved the predictive accuracy of aCI (Table 6). Therefore, the application of clinical risk factors in addition to SERPINE1 antibody levels may be necessary to establish a reliable diagnosis of aCI.

Acute ischemic stroke has high morbidity and mortality rates and is one of the leading causes of bed confinement. If prediction of the onset of stroke and early initiation of treatment can be achieved, the symptoms can be ameliorated or disappear; therefore, highly sensitive biomarkers must be identified. The SERPINE1 antibody level in combination with clinical factors is useful for predicting the risk of stroke, but further elaboration and development are needed to achieve higher sensitivity and specificity. The early clinical application of these biomarkers is expected to lead to the development of preventive treatments.

### Limitations

In this study, we discussed the potential of anti-SERPINE1 antibody level as a biomarker in stroke, but several limitations must be discussed. First, the prevalence of patients with TIA is low, and the small sample size may have resulted in a low positive predictive value and inconclusive results. In addition, the number of HDs was small in the prediction model, which may have resulted in inaccurate predictions. Second, because the blood samples were collected at the time of hospital admission, the date of stroke onset and admission differed among the patients. In addition, samples were obtained only from a Japanese population; samples from multiple ethnic groups should be analyzed for more conclusive findings. Finally, antibody levels alone had limited ability to predict ischemic stroke. The analysis of factors such as radiological findings and prognosis in addition to clinical factors could provide valuable insights; therefore, studies involving these factors should be conducted in the future.

## Supplementary Information

Supplementary Information.

## Data Availability

The data that support the findings of this study are available from the corresponding author upon reasonable request.
