# Supplementary material for: Serum anti-SERPINE1 antibody as a potential biomarker of acute cerebral infarction
Source: Sci Rep. 2021 Nov 5;11:21772. doi: 10.1038/s41598-021-01176-8 (PMC8571331; doi:10.1038/s41598-021-01176-8)
Supplement: Supplementary file 1 — Supplementary Information. [file 41598_2021_1176_MOESM1_ESM.docx]

**Supplementary Figure S1**

**
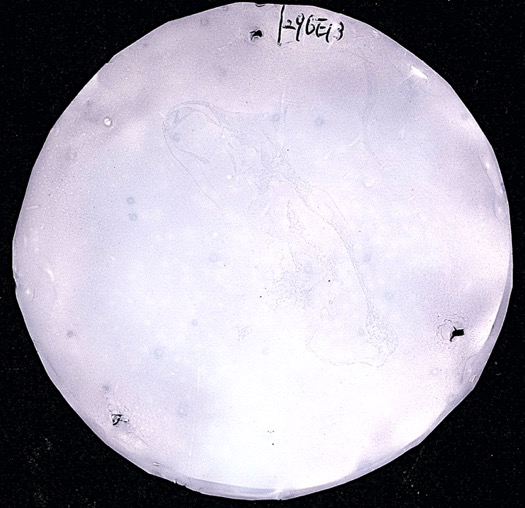
**

**Immunoscreening of transient ischemic attack antigens by SEREX**

Recombinant expression of cloning proteins was blotted onto nitrocellulose membranes and reacted with sera from sixteen TIA patients. Arrows indicate positive phage clones. Positive clones were re-cloned twice to obtain monoclonality.

**
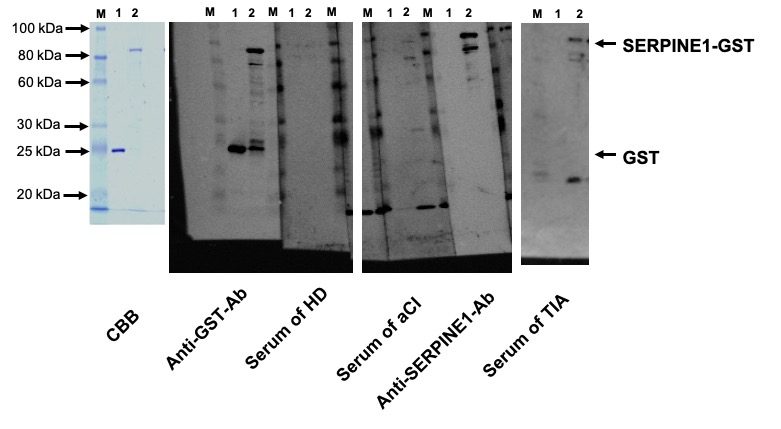
Supplementary Figure S2**

**Lane 1: GST**

**Lane 2: SERPINE-1 GST**

**Western blotting**

Full length of blots/gels presented in Fig.1 is shown. Affinity-purified glutathione-*S*-transferase (GST; lane 1), GST-tagged full length SERPINE1 proteins (lane 2), and molecular weight markers (lane M) were electrophoresed through sodium dodecyl sulfate-polyacrylamide (11%) gels. They were stained with Coomassie Brilliant Blue (CBB) or Western blotting using antibody or sera of participants.

**Supplementary Table S1.** Logistic regression analysis of the predictors of transient ischemic attack with SERPINE1 antibody levels as a continuous variable

|  | Univariate analysis | | | Multivariate analysis | | | |
| --- | --- | --- | --- | --- | --- | --- | --- |
|  | OR | 95% CI | *p* value | OR | 95%CI | *p* value |  |
| Age ≥60 years | 4.74 | 2.47 – 9.09 | **<0.0001** | 5.66 | 3.02 – 10.59 | **<0.0001** |  |
| Male sex | 0.94 | 0.46 – 1.91 | 0.8672 |  |  |  |  |
| HT | 3.09 | 1.66 – 5.77 | **0.0004** | 3.58 | 1.96 – 6.49 | **<0.0001** |  |
| DM | 4.72 | 1.87 – 11.91 | **0.0010** | 5.35 | 2.21 – 12.92 | **0.0002** |  |
| HL | 2.12 | 1.08 – 4.19 | **0.0296** | 1.83 | 0.95 – 3.52 | 0.0719 |  |
| CVD | 1.46 | 0.22 – 9.77 | 0.6938 |  |  |  |  |
| BMI ≥25 kg/m^2^ | 0.80 | 0.41 – 1.57 | 0.5219 |  |  |  |  |
| Smoking | 0.95 | 0.48 – 1.87 | 0.8748 |  |  |  |  |
| SERPINE1-Ab* | 1.01 | 0.99 – 1.03 | 0.2214 |  |  |  |  |

*: SERPINE1 antibody levels (per 100 counts) as continuous variable. The odds ratio indicates the value when a continuous variable change by one unit.

Univariate data with *p* values <0.05 were included in the multivariate analysis.

HT: hypertension, DM: diabetes mellitus, HL: hyperlipidemia, CVD: cardiovascular disease, BMI: body mass index, OR: odds ratio, CI: confidence interval, Ab: antibody.

**Supplementary Table S2.** Logistic regression analysis of the predictors of acute cerebral infarction with SERPINE1 antibody levels as a continuous variable

|  | Univariate analysis | | | Multivariate analysis | | | |
| --- | --- | --- | --- | --- | --- | --- | --- |
|  | OR | 95% CI | *p* value | OR | 95%CI | *p* value |  |
| Age ≥60 years | 13.8 | 8.66 – 21.95 | **<0.0001** | 13.4 | 8.63 – 20.75 | **<0.0001** |  |
| Male sex | 0.87 | 0.53 – 1.39 | 0.548 |  |  |  |  |
| HT | 5.45 | 3.47 – 8.56 | **<0.0001** | 4.9 | 3.19 – 7.49 | **<0.0001** |  |
| DM | 7.81 | 3.42 – 17.85 | **<0.0001** | 6.54 | 3.00 – 14.21 | **<0.0001** |  |
| HL | 0.58 | 0.32 – 1.01 | 0.0546 |  |  |  |  |
| CVD | 3.16 | 0.70 – 14.13 | 0.1315 |  |  |  |  |
| BMI ≥25 kg/m^2^ | 0.76 | 0.47 – 1.24 | 0.2796 |  |  |  |  |
| Smoking | 1.17 | 0.75 – 1.83 | 0.4819 |  |  |  |  |
| SERPINE1-Ab* | 1.02 | 1.00 – 1.03 | **0.0459** | 1.02 | 1.00 – 1.04 | **0.0312** |  |

*: SERPINE1 antibody levels (per 100 counts) as continuous variable. The odds ratio indicates the value when a continuous variable change by one unit.

Univariate data with *p* values <0.05 were included in the multivariate analysis.

HT: hypertension, DM: diabetes mellitus, HL: hyperlipidemia, CVD: cardiovascular disease, BMI: body mass index, OR: odds ratio, CI: confidence interval, Ab: antibody.

**Supplementary Table S3**

| **Variable** | **Spearman rank correlation coefficient (r)** | ***p* value** |
| --- | --- | --- |
| Age | -0.0356 | 0.5482 |
| Height | -0.0437 | 0.4621 |
| Weight | 0.0013 | 0.9832 |
| BMI | 0.0280 | 0.6399 |
| Right IMT | -0.0311 | 0.7065 |
| Left IMT | -0.0156 | 0.8503 |
| maximum IMT | 0.0307 | 0.7098 |
| AST | -0.0193 | 0.7933 |
| ALT | -0.0170 | 0.8176 |
| ALP | 0.1054 | 0.1652 |
| LDH | 0.0113 | 0.8789 |
| Tbil | 0.1537 | **0.0378** |
| CHE | 0.0316 | 0.7053 |
| γ-GTP | 0.0392 | 0.6027 |
| TP | -0.0318 | 0.6708 |
| ALB | 0.0015 | 0.9845 |
| BUN | -0.1012 | 0.1693 |
| Creatinine | -0.0434 | 0.5563 |
| eGFR | 0.0723 | 0.3460 |
| UA | -0.0561 | 0.5059 |
| AMY | -0.1516 | 0.0982 |
| T-CHO | 0.0175 | 0.8217 |
| HDL-C | -0.0864 | 0.3358 |
| TG | 0.0451 | 0.6031 |
| Na | 0.0144 | 0.8464 |
| K | -0.0850 | 0.2526 |
| Cl | 0.0306 | 0.6807 |
| CRP | 0.0215 | 0.8030 |
| WBC | 0.0908 | 0.2166 |
| RBC | -0.0280 | 0.7037 |
| HGB | -0.0084 | 0.9093 |
| HCT | -0.0157 | 0.8313 |
| PLT | 0.0862 | 0.2408 |
| Blood glucose | 0.1506 | **0.0448** |
| HbA1c | -0.0402 | 0.6160 |
| Smoking period | -0.0177 | 0.8100 |
| Alcohol frequency | -0.0700 | 0.4303 |
|  |  |  |

Correlation analysis between the SERPINE1 antigen levels and clinical features of the validation cohort. Correlation coefficient (*r*) and *p* values were calculated using Spearman correlation analysis. Significant correlations were marked in bold. We found no significant differences in antigen levels in terms of factors such as age and IMT, where significant differences in antibody levels were observed.

BMI: body mass index, IMT: intima-media thickness, AST: aspartate aminotransferase, ALT: Alanine aminotransferase, ALP: alkaline phosphatase, LDH: lactate dehydrogenase, Tbil: total bilirubin, CHE: choline esterase, γ-GTP: gamma-glutamyl transpeptidase, TP: total protein, ALB: albumin, BUN: blood urea nitrogen, eGFR: estimated glomerular filtration rate, UA: uric acid, AMY: amylase, T-CHO: total cholesterol, HDL-C: high-density lipoprotein cholesterol, TG: triglyceride, CRP: C-reactive protein, WBC: white blood cell count, RBC: red blood cell count, HCT: hematocrit, PLT: platelet count, HbA1c: hemoglobin A1c
